# Federated Learning for Thoracic Disease Classification Using Convolutional Neural Networks and Differential Privacy

**DOI:** 10.1049/htl2.70080

**Published:** 2026-05-02

**Authors:** Muhammad Zulqarnain, Syed Jawad Hussain, Muhammad Zeeshan Aslam, Ahsan Fiaz, Muhammad Islam

**Affiliations:** ^1^ Department of Computer Science Sir Syed CASE Institute of Technology Islamabad Pakistan; ^2^ Department of Computing Institute of Space Technology Islamabad Pakistan; ^3^ College of Science and Engineering James Cook University Cairns Queensland Australia

**Keywords:** convolutional neural networks (CNNs), differential privacy (DP), federated learning (FL), healthcare AI, medical imaging, multi‐label classification, remote healthcare, x‐ray diagnosis

## Abstract

Early diagnosis of thoracic diseases using chest x‐ray imaging remains a critical challenge, particularly in resource‐constrained healthcare environments where data sharing is restricted due to privacy concerns. Federated learning (FL) offers a decentralized solution by enabling collaborative model training without sharing sensitive patient data. However, integrating privacy‐preserving mechanisms such as differential privacy (DP) introduces additional challenges related to performance degradation and computational overhead. In this study, we present a unified FL framework for multi‐label thoracic disease classification using multiple convolutional neural network (CNN) architectures, including ResNet50, DenseNet169, EfficientNet variants and MobileNetV3. Unlike prior studies focusing on single‐model evaluation, this work provides a controlled comparative analysis under identical FL settings and investigates the impact of client scalability (5–10 clients) on model performance. Furthermore, we conduct a comprehensive empirical analysis of the privacy utility trade‐off by integrating DP with varying privacy budgets (*ε* = 1, 15 and 30). Experimental results on the CheXpert and NIH Chest x‐ray14 datasets demonstrate that the proposed EfficientNet‐B3‐based federated model achieves a mean AUC of 0.8027, while maintaining robustness across decentralized settings. The integration of DP leads to a predictable reduction in performance, with mean AUC ranging from 0.60 to 0.64, highlighting the inherent trade‐off between privacy and diagnostic accuracy. The findings emphasize the practical viability of FL for privacy‐sensitive medical imaging applications and provide insights into model selection, scalability and privacy configuration for real‐world deployment. The source code for this study is publicly accessible at https://github.com/Zulqarnain8‐8/FEDERATED_LEARNING_FOR_THORACIC_DISEASE_CLASSIFICATION.

## Graphical abstract

This study proposes a federated learning framework with differential privacy for thoracic disease classification from chest x‐rays in a multi‐label setting. Using the NIH Chest x‐ray14 and Stanford CheXpert datasets across multiple decentralized clients, five CNN models were evaluated, achieving an AUC of up to 0.8027 while ensuring patient confidentiality. The findings highlight a practical balance between performance and privacy, enabling secure and collaborative diagnostics for resource‐limited healthcare environments.

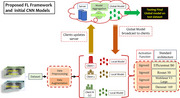

## 1 Introduction

Over the past two decades, the realm of machine learning (ML) has undergone a massive transformation [1]. Researchers perceive AI as a toolkit drawing from disciplines such as computation, mathematics, logic and biology; others define it as programmes capable of performing tasks [2]. DL is a specialized area within ML that consists of multiple layers, the number of which is known as ‘depth’. ML and DL are used in a wide variety of applications and are broadening their fields, such as image processing, data analysis, image classification and NLP [3]. For example, DL has enabled the development of transformer‐based models for medical imaging tasks [4], multi‐stage attention networks for image enhancement [5] and generative AI for multimodal motion synthesis [6]. Federated learning (FL) is an ML technique that enables numerous devices to work together in training a common model without disclosing their individual data to personalize recommendations that safeguard user information [7]. It offers a solution to the challenges posed by vast and decentralized datasets scattered across multiple devices [8]. Typically, remote healthcare facilities face challenges like a lack of advanced medical equipment and a shortage of healthcare professionals. Residents of these remote areas have limited access to medical services and facilities. Every year, lung diseases contribute significantly to the mortality rate, as evidenced by the outbreak of COVID‐19 in 2019 originating from Wuhan, China. An affordable and simple imaging method, chest x‐ray imaging, allows for the detection and screening of lung abnormalities resulting from infectious diseases like COVID‐19, pneumonia and tuberculosis (TB) [9]. Convolutional neural network (CNN) has become a vital technology, attracting considerable attention. Unlike older ML methods requiring extensive user involvement, the medical sector is increasingly turning to DL to support healthcare professionals in diagnostics [10]. These diagnosis processes facilitate timesaving for healthcare experts, but privacy and confidentiality concerns emerge when traditional AI methods handle medical data [8]. FL holds significant importance in the realm of medical image analysis, primarily due to its capability to address critical challenges faced in this domain [11]. Additionally, FL helps mitigate biases in medical datasets and ensures privacy compliance with strict regulations like the General Data Protection Regulation (GDPR), Personal Information Protection Law (PIPL) and the Health Insurance Portability and Accountability Act (HIPAA). As a result, it facilitates the development of robust and privacy‐preserving AI solutions for medical image analysis [11, 12]. Failure to adhere to data protection regulations can result in significant consequences, including hefty fines. For example, breaches under Article 83 of the GDPR may incur penalties of up to 20 million euros or 4% of total revenue for the fiscal year [12]. FL is still evolving; it has already demonstrated its utility in various sectors such as healthcare, transportation and finance. A significant portion of companies (32%) have either adopted or are planning to implement FL within the next few months to a year [2]. In light of these developments, the scope of this research will revolve around the application of FL in medical image analysis with a primary focus on chest x‐rays and the diagnosis of lung‐related diseases [13]. The study will aim to develop an FL framework tailored to the medical domain, emphasizing data privacy, collaboration and real‐world implementation in healthcare settings. The framework's design and evaluation will incorporate aspects related to privacy‐preserving, decentralized model training and practical deployment within healthcare facilities [8, 14]. The proposed study introduces an FL mechanism intended primarily for collaborative chest x‐ray processing across several decentralized healthcare clients. This study focuses on privacy‐preserving training by deploying neural networks at the client level and securely combining local models into a unified global model. The study intends to overcome significant issues in medical image diagnosis while adhering to data protection rules. The primary contributions of this study are summarized below.

### 1.1 Main Contributions

This study introduces an FL mechanism intended primarily for collaborative chest x‐ray processing across decentralized healthcare clients. The key contributions are as follows:

- This work presents a unified FL framework for multi‐label thoracic disease classification and provides a controlled comparative evaluation of multiple CNN architectures under identical FL settings, enabling fair performance analysis.
- Unlike prior studies focusing on single models or datasets, this study investigates the impact of client scalability (5 vs. 10 clients) on model performance in decentralized healthcare environments.
- A comprehensive empirical analysis of privacy–utility trade‐offs is conducted by integrating differential privacy (DP) with varying privacy budgets, highlighting its impact on diagnostic performance in medical imaging.
- The study emphasizes practical deployment considerations, including computational efficiency, communication constraints and applicability in resource‐limited healthcare settings.

## 2 Literature Review

DL models have provided a new avenue for medical professionals, as human performance is often limited in timely disease diagnosis [15]. As per the WHO, approximately 10 million individuals are affected by TB each year, resulting in 1.4 million deaths; about 65 million people suffer from chronic obstructive pulmonary disease (COPD), leading to roughly 3 million deaths annually [16]. COVID‐19 has triggered a severe global health emergency, and by 31 January 2021, the world had recorded over 103 million confirmed cases of the virus, with over 2.2 million deaths and around 26 million active cases [17]. This review seeks to provide a comprehensive overview of existing research in the domain of medical image analysis, with a specific focus on the application of FL and DP. It will delve into prior works’ methodologies and research outcomes, emphasizing the significance of this emerging ML paradigm in the context of medical image analysis and classification. At present, chest x‐rays stand as the most effective means for diagnosing pneumonia [18]. Using chest x‐rays for diagnosis exposes patients to lower levels of radiation than CT scans or MRIs, but interpreting x‐ray images poses distinct challenges [17]. A study based on the attention mechanism proposed a ResNet attention technique with 95% accuracy for classifying chest x‐rays into two classes [19]. A multi‐head self‐attention ResNet model was used for the classification of chest x‐rays and achieved an ACC of 95.52% [20].

The success of deep learning is often constrained by data scarcity, particularly in specialized domains like healthcare and agriculture where large labelled datasets are infeasible. The comprehensive survey by Alzubaidi et al. [44] systematically defines this challenge, categorizing the limitations of traditional models when trained on insufficient data. The authors synthesize a wide range of solutions, including data augmentation, transfer learning, few‐shot learning and generative models, while also providing practical tips for implementation across various applications. Their work serves as a foundational reference for researchers seeking to develop robust deep learning models under severe data constraints.

A DL approach is introduced to classify chest x‐ray images into four categories and attains the highest score of 96.13% [21]. Another study presents an approach for classifying chest x‐rays using Xception and VGG models; VGG‐16 outperformed the Xception model [22]. A dataset of 1000 CT scan images was used to classify various lung cancer types; VGG‐16 achieved 77.63% ACC [23]. A lightweight attention mechanism based on ResNet50 was proposed for the classification of breast cancer. Leaky ReLU is used and outperforms other models with an ACC of 0.987 [24]. L2 regularization and NIH chest x‐ray dataset, along with EfficientNetB7 GAP, outperformed others with a validation accuracy of 96.22% [10]. CheXNet is a 121‐layer CNN used with weighted binary cross‐entropy loss and the Adam optimizer to perform binary classification, achieving an AUROC of 0.7680 in pneumonia detection [18]. The five‐fold cross‐validation technique was implemented using five different pre‐trained models. Among them ResNet‐50 achieved an average ACC of 96.1% [17].

A study proposed a hybrid DL algorithm (HDLA) in which ResNet50 served the role of feature extraction, followed by scaling using min–max normalization, including random forest (RF) and support vector machine (SVM). HDLA outperformed others with an accuracy of 98.99% in less time [25]. Chest disease classifier network was compared with ResNet50, VGG19 and InceptionV3; the model outperformed others with an AUC score of 0.9953 [15]. DenseNet‐121 model is trained with a batch size of 8 and initial learning rate of 0.0001 for 20 epochs using Adam optimization and achieves a mean AUC of 0.8537 [26]. EfficientNet B0 was tested for its ability to classify COVID‐19, pneumonia and TB; it achieved an impressive accuracy score of 0.99 [9]. The MobileNetV2 model used for classifying lung disease delivers an accuracy of 55.47% [27]. The pre‐trained model was fine‐tuned for the detection of multiple diseases from a single chest x‐ray dataset and cross‐entropy using the FL method. The federated approach was compared to existing methods, and the results showed that the federated model achieved higher performance in predicting multi‐labelled chest diseases [28].

Four CNN models, MobileNetV2, ResNet18, ResNeXt and COVID‐Net, were tested with the FL framework and without it; the results indicated that ResNet18 exhibited the fastest convergence and achieved the highest accuracy of 91.26% [19]. In 2005, DARPA's Information Processing Technology Office (IPTO) introduced a new concept called transfer learning. This involves systems being able to utilize knowledge and skills acquired from previous tasks to assist with new tasks [29]. Recently, Google introduced the concept of ‘federated ML’ or ‘FL’, which is the fundamental concept of avoiding transferring user data between devices to ensure privacy [14]. FL has also shown promising results in domains like vehicular edge computing, where it enhances predictive content caching and privacy preservation [30]. The main aim of FL is to ensure the privacy of participating companies, organizations and numerous individuals, but various methods of attacks are available to target the model, such as data poisoning attacks and model inversion attacks. Privacy techniques, data encryption and homomorphic encryption are susceptible to various vulnerabilities, such as model inversion attacks. To overcome these challenges, numerous methods, such as DP, model aggregation and cryptographic methods, are used to protect the data in FL systems [2]. DP, as a modern approach to privacy, ensures that the outcomes of a model are not significantly affected by the presence or absence of any individual dataset [2]. It adds noise to the model's outputs and is characterized by two key parameters: 𝜖 (epsilon) and *𝛿* (delta). Epsilon indicates how much information about the dataset can be inferred from the model's output, whereas delta measures the probability of accidental leaks of additional information [31].

Recent works have addressed various aspects of FL, as illustrated in Table 1, but there's still a gap in the literature regarding implementing this approach in medicine and healthcare. The application of FL in healthcare has been explored, often with a focus on specific areas like electronic health records or the network of medical devices, often overlooking the important field of medical imaging [8]. Today, publicly available datasets have been recognized as extremely effective in the realm of DL models in tasks such as image recognition, NLP and medical disease diagnosis. Several datasets are publicly available for x‐ray image classification tasks [16, 32].

- **Montgomery County (MC) and Shenzhen Datasets**: MC contains 138 frontal chest x‐ray images, and the Shenzhen dataset comprises 662 x‐rays [33].
- **Japanese Society of Radiological Technology (JSRT) Dataset**: A widely used dataset for lung nodule detection and classification [16].
- **Open‐I Dataset**: Includes 3996 chest radiology reports and associated images [34].
- **Chest x‐ray14 Dataset**: Contains 112,120 frontal chest x‐rays from 30,805 patients with 14 labelled thoracic diseases [35].
- **CheXpert Dataset**: Comprises 224,316 images with 14 labelled observations from 65,240 patients [36].
- **MIMIC‐CXR Dataset**: Includes a total of 377,110 chest x‐ray images from 65,379 patients [37].
- **PadChest, PLCO and VinDr Datasets**: PadChest includes 160,868 images from 67,000 patients, PLCO has 185,421 images from 56,071 patients and VinDr consists of 18,000 x‐rays [16, 32].

**TABLE 1:** Summary of various DL techniques.

Author	Models and techniques	Achievements	Limitations
Singh et al. [38]	ResNet attention	Prediction of pneumonia	Limited dataset
Khan et al. [21]	EfficientNetB1, NasNet‐Mobile and MobileNetV2	Detection of viral lung diseases	Integrated datasets from multiple sources
Ayan and Ünver [22]	Xception and VGG‐16	Diagnosis of pneumonia	Dependence on a limited, single dataset
Vij and Kaswan [23]	VGG‐16	Prediction of lung cancer	Relatively low accuracy due to a very limited dataset
Liu [24]	ResNet	Breast cancer classification	Insufficient data privacy for sensitive information
Hong et al. [10]	EfficientNet‐B7, GAP and preprocessing	Pneumonia, pneumothorax and tuberculosis	High computational cost, limited to NIH dataset
Rajpurkar et al. [18]	CheXNet—121‐layer DenseNet	Classification of pneumonia	Based on a single dataset and model
Narin et al. [17]	ResNet, InceptionV3	Classification of COVID‐19 and viral pneumonia	Focus only on binary classification
Farhan and Yang [25]	HDLA with ResNet	Pneumonia and COVID‐19	Lack of attention mechanism
Malik et al. [15]	CDC Net	COVID‐19, pneumothorax, pneumonia, lung cancer and tuberculosis	High computational complexity, lack of patient privacy
Zhao et al. [26]	DenseNet‐121 with CBAM	Multi‐labelled disease classification	Lack of patient privacy, moderate computational complexity
Sultana et al. [9]	EfficientNet B0	Classification of COVID‐19, pneumonia and tuberculosis	Lack of patient privacy and limited dataset details
Vanga et al. [27]	DenseNet121, InceptionV3 and ensemble	Adenocarcinoma, large cell carcinoma, COVID‐19, pneumonia and tuberculosis	Relatively low accuracy
Priya and Peter [28]	DenseNet‐121 + FL	Multiple diseases	Limited to a single dataset and model
Liu et al. [19]	MobileNetV2, ResNet18, ResNeXt and COVID‐Net + FL	Prediction of COVID‐19 and pneumonia	Single dataset, limited class prediction and lack of image enhancement

Abbreviation: FL, federated learning.

Recent advancements in computer‐aided diagnosis (CAD) using ML and DL models like ResNet, DenseNet, MobileNet, EfficientNet and VGG have improved chest x‐ray disease classification accuracy. Enhanced with attention mechanisms and transfer learning, these models show stronger results but often rely on centralized methods that risk privacy. FL offers a privacy‐preserving alternative, though recent research studies are mostly limited to a few datasets and diseases. Although FL has shown promise in healthcare, especially for EHRs and medical devices, its use in medical imaging remains limited. Challenges like data heterogeneity, model attacks and privacy issues persist, but techniques like DP can help FL evolve for large‐scale imaging.

## 3 Proposed Methodology

Unlike prior studies that primarily focus on maximizing classification accuracy in centralized settings, this work emphasizes the practical challenges of deploying AI models in real‐world healthcare environments, where data privacy, decentralization and limited computational resources are key constraints. Therefore, the primary objective is not to outperform all existing methods in terms of accuracy, but to provide a systematic analysis of model performance under federated and privacy‐preserving conditions. This perspective is critical for bridging the gap between research and real‐world clinical deployment.

This section explains the various approaches implemented for classifying x‐rays of 14 thoracic classes. The primary focus is the evaluation of CNN models using privacy‐preserving approaches to achieve the research objectives. The literature review has been carried out extensively, which shows that CNN models performed well on various datasets in a centralized method. Centralized and non‐private models present threats to patient secrecy as they gather sensitive medical data from various sources and concentrate it in one central repository for examination. In this research, our focus revolves around the classification of thoracic diseases, a crucial aspect of medical diagnosis and treatment. To achieve this, initially, four different CNN models (well‐known for their efficiency in image classification tasks) were evaluated using an FL environment, namely, ResNet‐50, DenseNet‐169, EfficientNet‐B0 and MobileNet‐V3 Large.

### 3.1 Dataset

Dataset plays a significant role in DL, but the same is limited due to privacy. In the case of the medical field, it is much more difficult to acquire a dataset with accurate labels. Many datasets have been reviewed; among these, the largest datasets are MIMIC (with electronic health records), CheXpert and NIH Xray14, which are publicly available. CheXpert and NIH x‐ray 14 are famous for their comprehensive collections of chest x‐ray images. These have been widely utilized in numerous studies, providing rich resources for conducting thorough analyses into thoracic disease classification. In this research, both the CheXpert and NIH x‐ray datasets have been utilized in different experiments for training of various CNN models within the framework of FL.

### 3.2 Models

The CNN models have demonstrated exceptional well in the field of x‐ray classification and disease prediction. Numerous studies have utilized various CNN architectures by implementing training from scratch and pre‐trained models with different fine‐tuning strategies. Following this trend, initially, our research plans to use four specific CNN models which are ResNet50 (addressing vanishing gradient problem), EfficientNetB0 (fewer parameters and optimizing computational efficiency), DenseNet169 (promoting feature reuse and interpretability) and MobileNetV3Large (emphasizing lightweight architecture and lower computational requirements) for the classification of 14—thoracic disease. All the models utilize pre‐trained weights available in PyTorch and adapt these for specific tasks. We initially modified the classifier layer of the pre‐trained models and included the sigmoid activation function (often used in the output layers of DL architectures for predicting probabilities in outputs) [16]. This transformation is particularly useful for multi‐label classification tasks, ensuring that the output dimensionality matches the desired number of classes while also enabling the models to generate probabilities for each class independently.

### 3.3 Proposed Framework With FL

**FIGURE 1:**
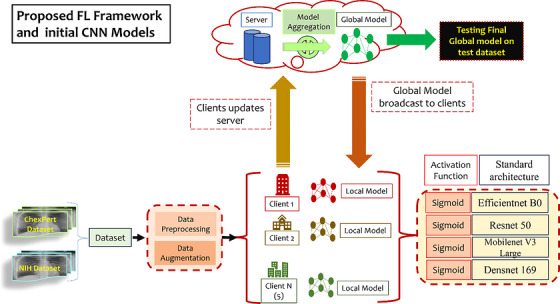
Proposed framework—(Experiments 1 and 2). CNN, convolutional neural network; FL, federated learning.

The proposed framework is based on FL, a decentralized training approach that has demonstrated significant performance and privacy of patient data. One key element of FL is federated averaging, where the global model is initialized and shared with various clients. Clients then train the global model on their localized datasets, and after each communication round, model updates are aggregated by computing a weighted average of the clients’ parameters. The steps/algorithm involved in this complete process are illustrated in Figure 1, and the algorithm is shown in Table 2.

**TABLE 2:** Algorithm.

1. 2. 3. 4. 5. 6. 7. 8. 9.	Load data Preprocess the data Normalize the data Apply data augmentation techniques Create custom datasets from the processed data Define the total number of clients: C Split the dataset into subsets S₁, S_2_,…, S_C for each client. Initialize the global model: G₀ For each communication round i = 1, 2,…, do: a. Select a fraction F of clients: C_f ← max(F × C, 1) b. Randomly select a set of participating clients: C_i ⊆ C_f c. For each client c ∈ C_i (in parallel), perform: G^c^_{i+1} ← ClientUpdate(c, G_i_) d. Aggregate updates from all participating clients: G_{i+1} ← ∑ (n_c / n) × G^c^_{i+1} for all c ∈ C_i where n_c = data size of client c n = total data size across selected clients
Return the final global model G	

### 3.4 Experiments

**FIGURE 2:**
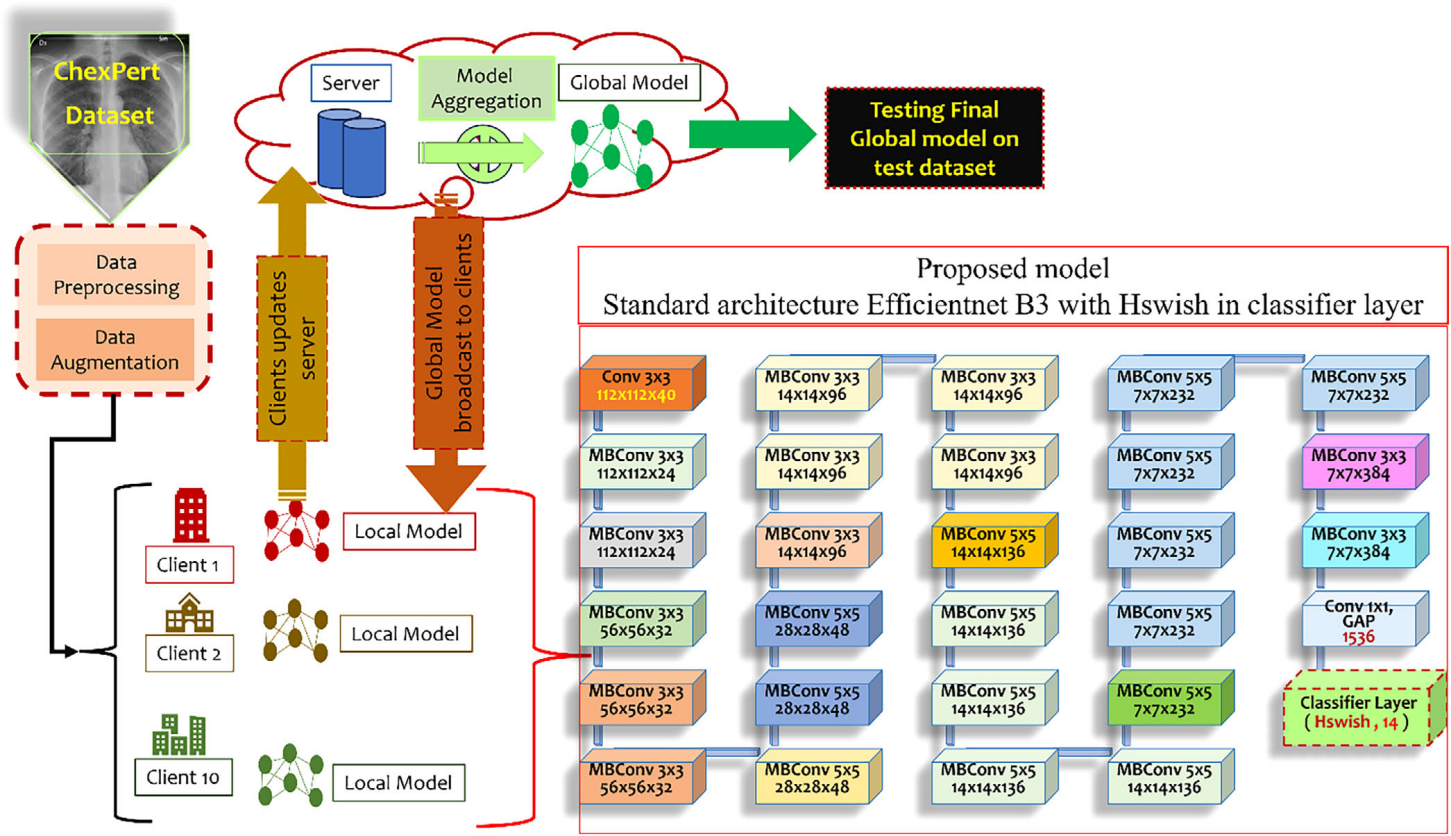
Proposed model and FL framework.

In the experimental part, CNN models are executed using the methodologies outlined in various related works. CNN models are run on the Kaggle platform, which provides access to GPUs such as Tesla P100, featuring 15.84 GB of GPU memory utilizing a combination of 3584 CUDA cores with an Intel Xeon CPU @ 2.00GHz and a total RAM capacity of approximately 25 GB. The environment supports Python version 3.10.12, packaged by conda‐forge and PyTorch version 2.0.0. These hardware and software specifications contribute to the effective training and evaluation of the selected models on both the NIH x‐ray 14 and CheXpert datasets. Three experiments were conducted on various parameters using the FL framework, each of which plays a significant role in optimizing model performance and ensuring its adaptability to the dataset. A fourth experiment was conducted using the framework of FL with DP. This integration aimed to enhance privacy while evaluating model performance at different privacy levels. Therefore, further refinement of the model is imperative for heightened performance. Subsequently, an additional experiment (Experiment 3) was conducted utilizing another variant of EfficientNet, ‘B3’, on the CheXpert dataset to evaluate deeper and explore its potential. The hyperparameters for this experiment have been thoroughly discussed and are detailed in Table 3, whereas the model architecture is depicted in Figure 2.

**TABLE 3:** Hyperparameter settings for Experiments 1–4.

Hyperparameters	Experiments 1 and 2	Experiment 3	Experiment 4
Batch size	32	64	60
Epochs	7	3	4
Comm rounds	3	7	3
Learning rate	0.001	0.001	0.001
Optimizer	Adam	AdamW	AdamW
Activation function	Sigmoid	Hswish	Hswish
Loss function	BCE loss	BCEwithlogits	BCEwithlogits
Scheduling technique	—	ReduceLRonPlateau	ReduceLRonPlateau
Epsilon	—	—	1, 15, 30
Delta	—	—	1∕(Client dataset)

### 3.5 Proposed Method With DP

DP involves enhancing data privacy through the addition of random noise, thereby increasing the difficulty of accessing individual information within a dataset [17]. In this study, we employed Opacus, a powerful library designed for training PyTorch models with DP. It uses differentially private stochastic gradient descent (DP‐SGD). This approach emphasizes injecting noise into the parameter gradients used by the model, rather than altering the training data directly. In this way, it prevents the model from memorizing individual training examples while enabling effective learning from the collective data. Opacus primarily applies per‐sample DP, ensuring that each data point maintains privacy protections [15]. Adding noise requires careful calibration, as excessive noise can distort the signal, whereas insufficient noise may not adequately protect privacy. Opacus employs the Gaussian mechanism, which relies on two important parameters: A noise multiplier and a constraint on the maximum gradient size, by carefully adding noise based on these parameters, Opacus ensures that DP is maintained during training [8]. BatchNorm calculates statistics (mean and variance) across a batch of data, which makes each sample's normalized value dependent on others in the batch. This dependency violates the fundamental principle of DP, which requires that each sample's contribution to the final model's output be independent of other samples. To address this, DP supports other normalization methods, such as GroupNorm. The Module Validator in Opacus checks neural network modules, like those in EfficientNet B3, to ensure that they are compatible with DP and replaces incompatible layers, such as BatchNorm, with GroupNorm.

## 4 Experiments and Results

The results section of this thesis provides a comprehensive analysis of the performance of five CNN models within the privacy‐preserving framework across four experiments. The effectiveness of each CNN model was assessed within collaborative learning scenarios utilizing two different datasets. Experiments 1 and 2, which involve ResNet‐50, EfficientNet‐B0, DenseNet‐169 and MobileNet‐V3Large, were conducted on both datasets. In Experiments 3 and 4, a variant of EfficientNet ‘B3’ was utilized for the classification of thoracic diseases from x‐ray images with FL and DP.

### 4.1 Evaluation

Evaluation is the process of assessing the performance of any system, process or model against predefined criteria, principles, or standards. In the DL, evaluation refers to the systematic assessment and valuation of the performance of a neural network model. DL models are evaluated using various performance metrics, depending on the nature of the task [20]. The evaluation of classification models involves several key metrics, each providing unique insights into the model's performance. A few terminologies related to evaluation metrics are as follows [38]:

- **True positive (TP)** refers to a sample that the model correctly identifies as positive, consistent with the actual ground truth.
- **False positives (FPs)** are samples that the model incorrectly identifies as positive, different from the actual ground truth.
- **True negative (TN)** indicates a sample that the model correctly identifies as negative, in alignment with the actual ground truth.
- **False negatives (FNs)** are samples that the model incorrectly identifies as negative, opposing the actual ground truth.
- **True positive rate (TPR)**. It is also known as ‘sensitivity’ which measures the proportion or ratio of actual positive instances correctly identified by the following model.
(1) $$\mathrm{TPR}=\;\frac{\mathrm{TP}}{\mathrm{TP}+\mathrm{TN}}$$
- **False positive rate (FPR)**. It is the ratio of the actual negatives which are mistakenly classified as positives.
- **True negative rate (TNR)**. It is also known as specificity, which indicates the proportion of actual negatives correctly identified by the model.
(2) $$\mathrm{TNR}=\;\frac{\mathrm{TN}}{\mathrm{TN}+\mathrm{FP}}$$

Hence, TPR (sensitivity) denotes the proportion of actual positives (unhealthy condition of a person) correctly identified by the model. FP rate quantifies the proportion of actual negatives mistakenly classified as positives (healthy individuals wrongly identified as having the disease). Specificity (TNR) represents the proportion of actual negatives (condition of a healthy person) correctly identified by the model.

### 4.2 AUC–ROC

The AUC–ROC is a widely recognized metric in medical imaging analysis and performance evaluation of binary classification tasks, especially within CAD systems. It evaluates a model's performance by considering both its correct and incorrect predictions [21]. The ROC curve acts as a tool to evaluate binary classification tasks by illustrating the balance between TPR and FPR across various thresholds. A higher AUC score indicates better performance of a model in distinguishing between positive and negative classes. For classification tasks, the AUC–ROC curve is a go‐to metric that is vital for evaluating the performance of any classification model, especially in multi‐labelled/class scenarios [39].

#### 4.2.1 Analysis Experiment 1

In our Experiment 1 with CNN models and ChexPert dataset, we adopted a standardized approach and customized the classifier to include a ‘sigmoid’ activation to ensure that the model directly generates probabilities for each class alongside 5 clients. Throughout our experimentation, we focused on employing the standard architecture of each CNN model within the context of FL methodology, with a particular emphasis on federated averaging techniques to evaluate model performance. Finally, to assess the performance of each CNN model, we conducted a final test on a separate test dataset. The class‐wise AUC scores of each related to CheXpert datasets and Experiment 1 are illustrated in Table 4. Overall, in terms of AUC scores, MobileNetV3 Large and EfficientNetB0 performed better comparatively, with mean AUC values of 0.7589 and 0.7522, respectively. Both models exhibited strong performance across multiple disease classes in this experiment, surpassing deeper models in comparison.

**TABLE 4:** AUC score on CheXpert dataset.

Classes	EfficientNet B0	ResNet50	MobileNet V3 Large	DenseNet 169
No finding	0.8804	0.8626	0.8695	0.8805
Enlarged cardio	0.5860	0.5786	0.5674	0.5267
Lung opacity	0.6715	0.6280	0.7051	0.6622
Lung lesion	0.7504	0.7721	0.7479	0.7013
Fracture	0.6913	0.7015	0.7770	0.7461
Support devices	0.8742	0.8244	0.8467	0.8613
Pleural effusion	0.8626	0.8611	0.8697	0.8486
Consolidation	0.7534	0.6871	0.7034	0.6855
Pneumonia	0.7023	0.6140	0.7411	0.6944
Oedema	0.8150	0.7721	0.7964	0.8142
Atelectasis	0.6524	0.6229	0.6791	0.6566
Pneumothorax	0.7684	0.7568	0.8374	0.7881
Cardiomegaly	0.8130	0.7684	0.8358	0.7809
Pleural thickening	0.7103	0.7026	0.6479	0.7004
**AUC mean**	**0.7522**	**0.7251**	**0.7589**	**0.7390**

#### 4.2.2 Analysis Experiment 2

In our Experiment 2 with CNN models, we adopted the same approach as Experiment 1 on the NIH x‐ray 14 dataset alongside 5 clients. Various datasets exhibit varying quality, with images differing across datasets. Therefore, evaluating models only on single datasets may not offer optimal insight into their performance. When operating within an FL framework, models should be trained using various datasets to ensure the robustness of each dataset. In this experiment, we employed the NIH x‐ray 14 dataset to assess the robustness of each model within the FL framework, using a range of models to enhance experimentation. The class‐wise AUC scores of each related to NIH x‐ray 14 datasets and Experiment 2 are illustrated in Table 5. Overall, both the EfficientNetB0 and DenseNet169 models exhibited strong performance across a range of disease classes, achieving mean AUC scores of 0.7533 and 0.7620, respectively. This suggests the effectiveness and reliability of both models in accurately classifying thoracic diseases.

**TABLE 5:** AUC score on NIH x‐ray 14 dataset.

Classes	EfficientNet B0	ResNet50	MobileNet V3 Large	DenseNet 169
No finding	0.7770	0.7644	0.7837	0.7967
Enlarged cardio	0.6826	0.6957	0.7131	0.7162
Lung opacity	0.6738	0.6262	0.6452	0.6752
Lung lesion	0.7908	0.8033	0.8073	0.8046
Fracture	0.7058	0.6954	0.7131	0.7301
Support devices	0.7982	0.7297	0.7818	0.7918
Pleural effusion	0.8194	0.8031	0.8319	0.8405
Consolidation	0.7106	0.6973	0.7032	0.7230
Pneumonia	0.6651	0.6471	0.6712	0.6764
Oedema	0.6984	0.6515	0.6489	0.7071
Atelectasis	0.7418	0.7426	0.7393	0.7658
Pneumothorax	0.8460	0.7722	0.7600	0.8224
Cardiomegaly	0.7600	0.7371	0.7255	0.7500
Pleural thickening	0.8760	0.7951	0.7643	0.8676
**AUC mean**	**0.7533**	**0.7258**	**0.7349**	**0.7620**

**FIGURE 3:**
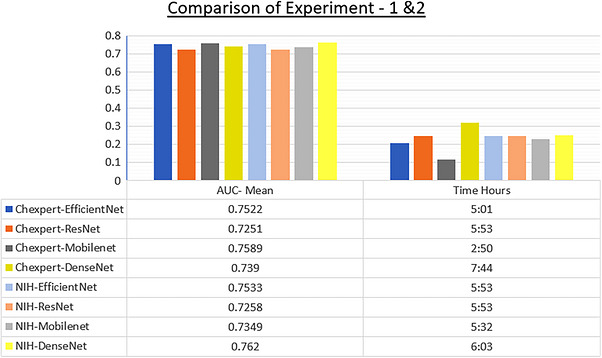
Comparison of AUC mean and training time.

The assessment of various CNN models using different datasets provided significant insights and knowledge. Across both experiments, we have compared the models in terms of mean AUC and time efficiency to determine their performance in FL settings. In Experiment 1, EfficientNet and MobileNet demonstrated better mean AUC performance. In Experiment 2, conducted on the NIH x‐ray 14 dataset, DenseNet169 outperformed other models with a mean AUC of 0.762. However, it incurred the longest training time of 6 h and 3 min. Similarly, when trained on the CheXpert dataset, DenseNet169 also had the longest training time of 7 h and 44 min. Overall, considering AUC score and time efficiency, MobileNetV3 Large has the best time efficiency, followed by EfficientNet B0 with a better AUC score on both datasets, as illustrated in Figure 3.

#### 4.2.3 Analysis Experiment 3

**FIGURE 4:**
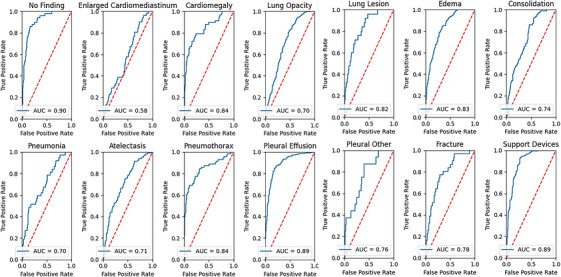
ROC of proposed model (efficient net B3).

In this experiment, our main focus was utilizing a variant of EfficientNet, which is B3.3, within the framework of FL. We specifically emphasized the use of federated averaging to assess the effectiveness of the model. We employed a pre‐trained EfficientNet B3 model with an additional Hswish activation function in the classifier layer. Throughout this experiment, the federated averaging approach was employed with 10 selected clients to facilitate collaborative learning. This modification aimed to evaluate the performance of FL with a larger client pool, reflecting real‐world scenarios where small hospitals in remote areas lack access to accurately labelled datasets and specialized radiologists for the timely diagnosis of diseases. The experimental AUC scores for each class are presented in Table 6 and illustrated in Figure 4, revealing a mean.

**TABLE 6:** Federated learning (FL) with differential privacy (DP) (class‐wise and mean AUC score).

Classes	𝜖 = 1, 𝜎 = 0.7238	𝜖 = 15, 𝜎 = 0.3575	𝜖 = 30, 𝜎 = 0.2870	Our FL (EfficientNet B3)
Enlarged cardiomediastinum	0.4921	0.5515	0.5534	0.6208
Cardiomegaly	0.5730	0.5916	0.5957	0.8346
Lung opacity	0.5906	0.6016	0.6212	0.7259
Lung lesion	0.5457	0.5917	0.6189	0.8377
Oedema	0.6686	0.7211	0.7418	0.8466
Consolidation	0.5971	0.6046	0.6313	0.7529
Pneumonia	0.5051	0.4565	0.4648	0.7532
Atelectasis	0.6177	0.6099	0.6143	0.6978
Pneumothorax	0.4904	0.5436	0.5900	0.8539
Pleural effusion	0.6677	0.6952	0.7344	0.8939
Pleural other	0.6187	0.6591	0.6220	0.7822
Fracture	0.6202	0.6042	0.6211	0.8391
Support devices	0.6414	0.7123	0.7425	0.8921
No findings	0.7725	0.8006	0.8269	0.9066
**AUC mean**	0.6000	0.6245	0.6413	**0.8027**

**FIGURE 5:**
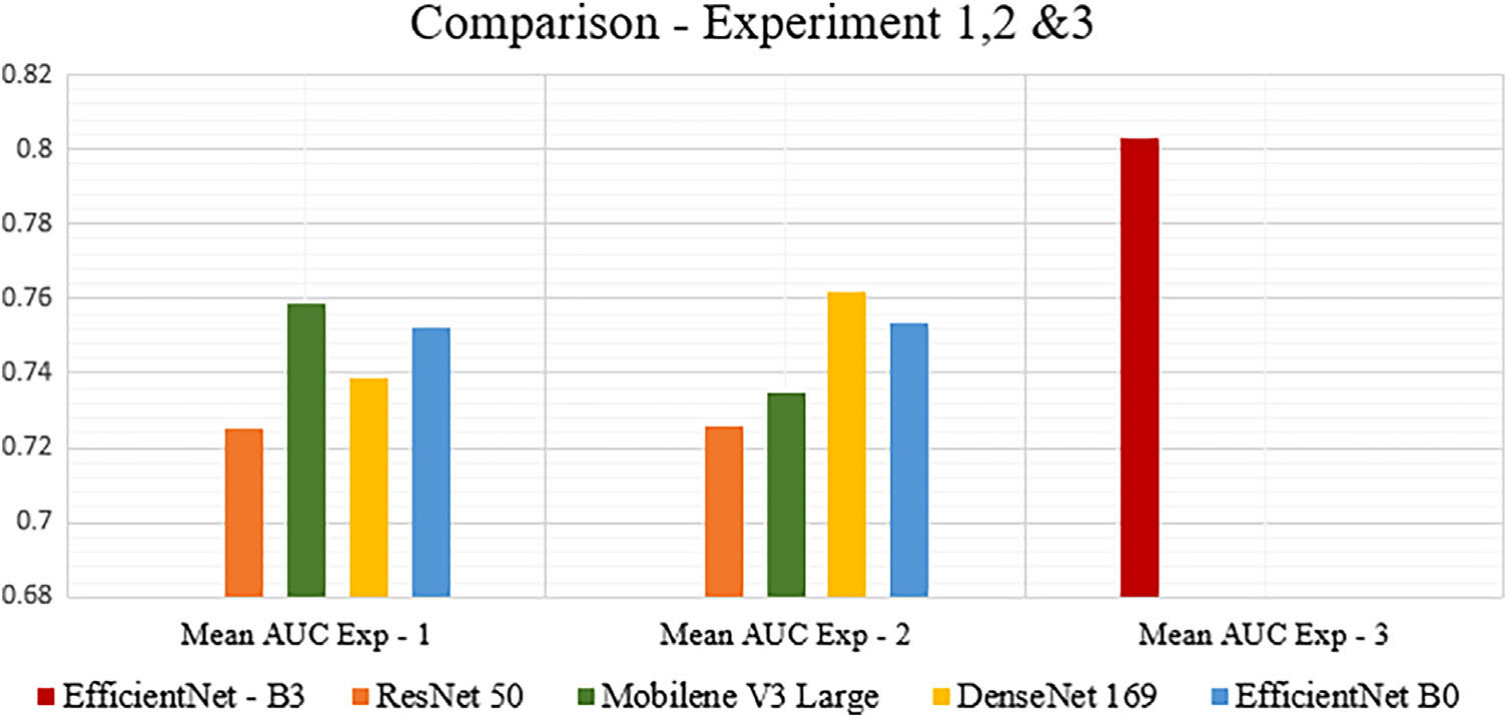
Comparison Experiments 1–3.

AUC score is 0.8027. This outcome suggests that expanding the number of clients from 5 to 10 does not significantly diminish the performance of the collaboratively trained model. Using the Hswish activation function instead of the sigmoid activation function in the classifier layer improves performance. Implementation of BCE with logit loss, along with the ReduceLROnPlateau learning rate scheduler, leads to better outcomes as compared to the first and second experiments. Additionally, when comparing this experiment to Experiments 1 and 2, this experiment demonstrated an improved AUC of 0.8027. It surpassed the previous experiments with an increased margin of +0.0407 AUC, leading to better classification across all classes, as illustrated in Figure 5.

#### 4.2.4 Analysis Experiment 4

In this experiment, we integrated DP with the proposed framework (FL with EfficientNet B3). We compared the AUC scores with our previous experiment. The experimental AUC scores for each target value of epsilon (𝜀 = 1, 15 and 30) and noise multiplier (𝜎) across each class are presented in Table 6.

**FIGURE 6:**
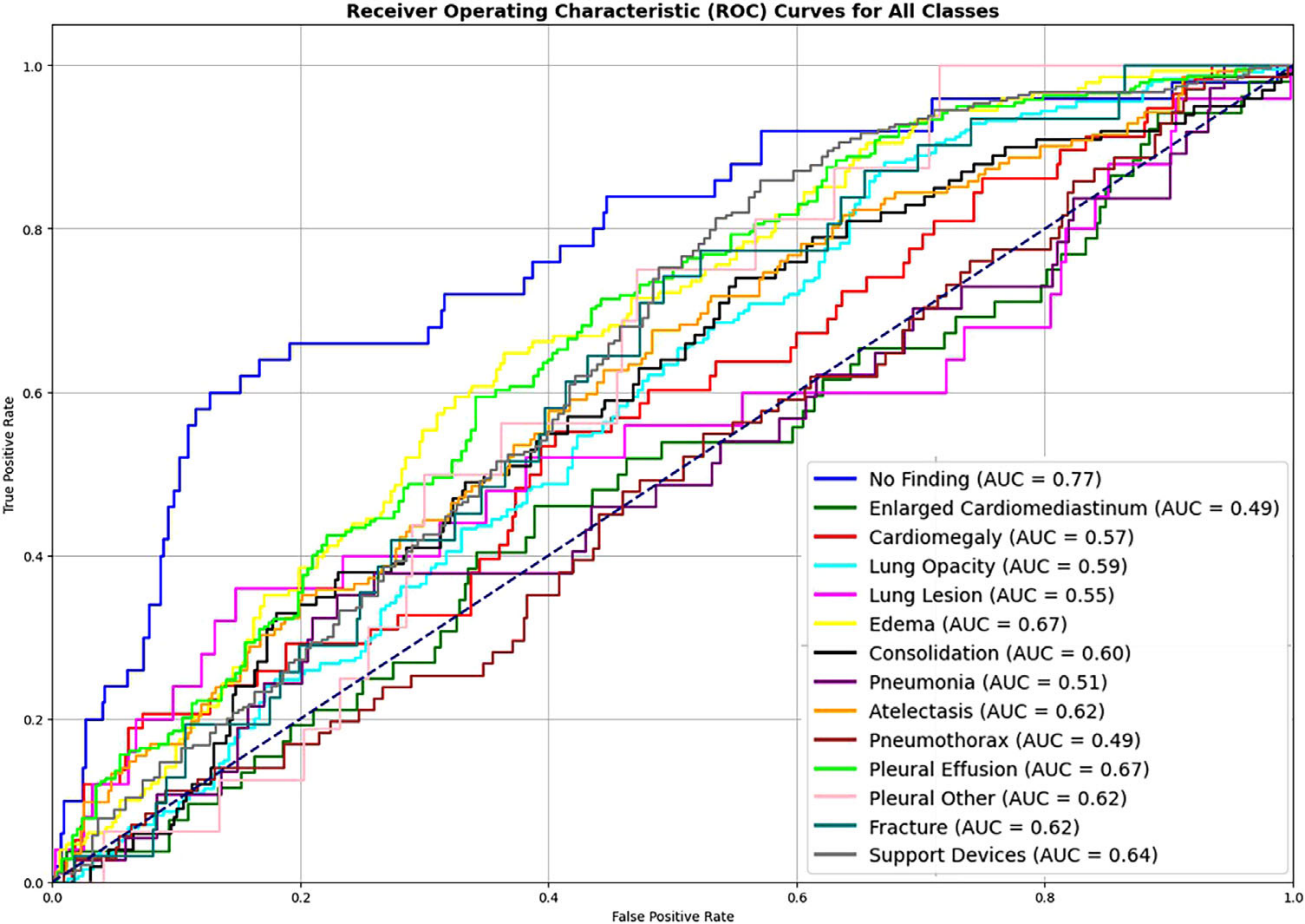
Class‐wise characteristic curves: 𝜖 = 15, 𝜎 = 0.3575 (indicates moderate privacy).

**FIGURE 7:**
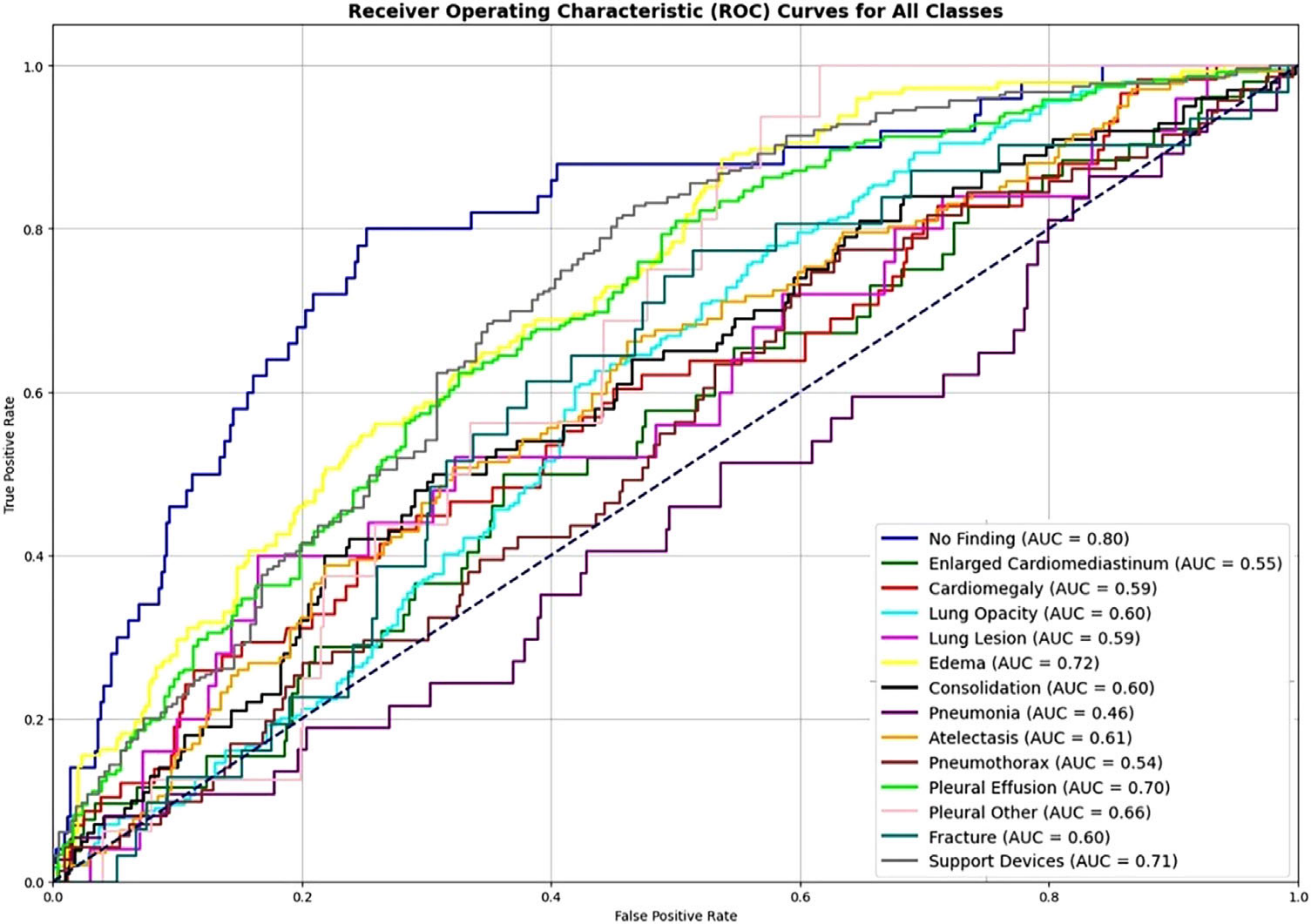
Class‐wise characteristic curves: 𝜖 = 15, 𝜎 = 0.3575 (indicates high privacy).

**FIGURE 8:**
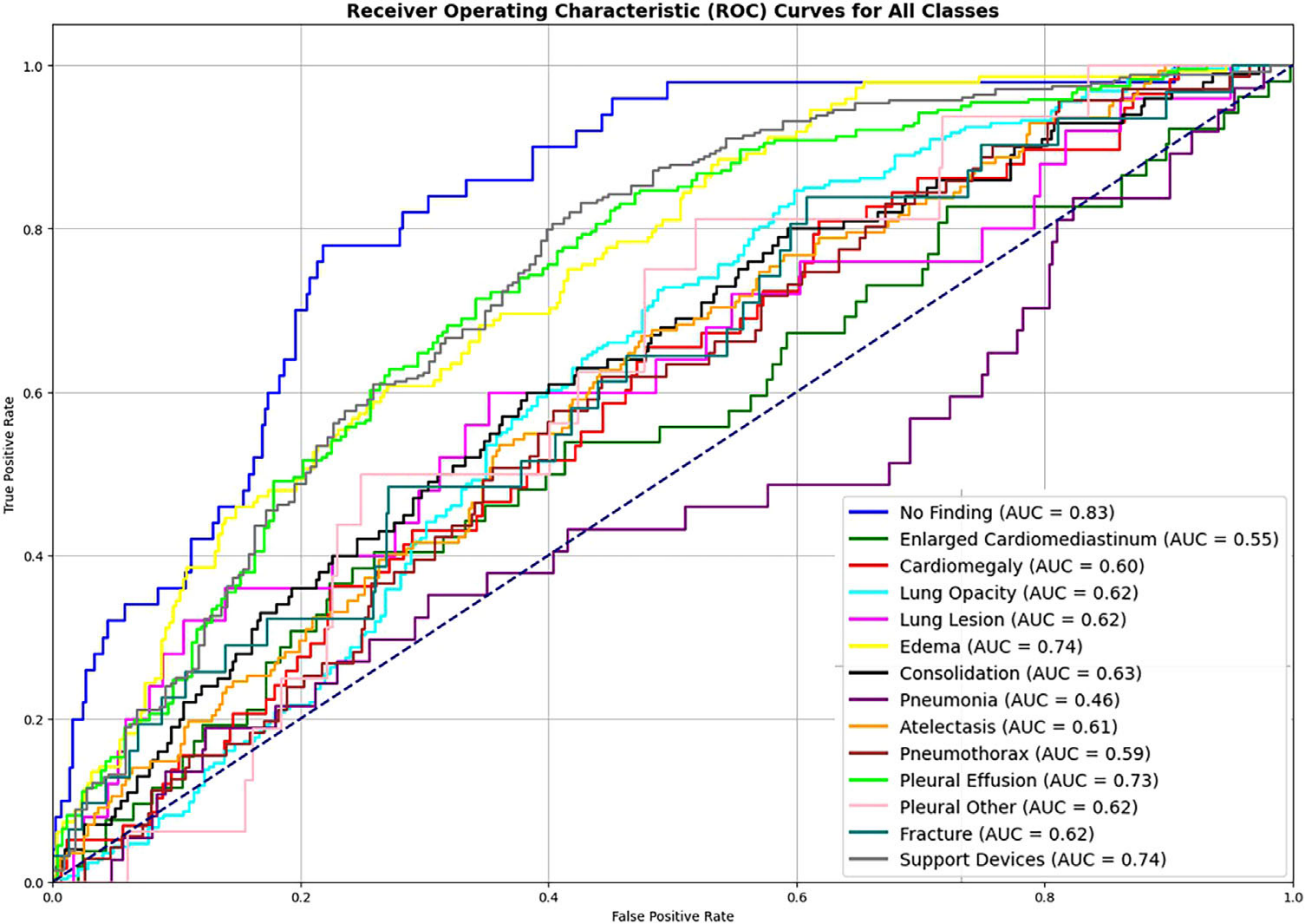
Class‐wise characteristic curves: 𝜖 = 30, 𝜎 = 0.2870 (indicates low privacy).

**FIGURE 9:**
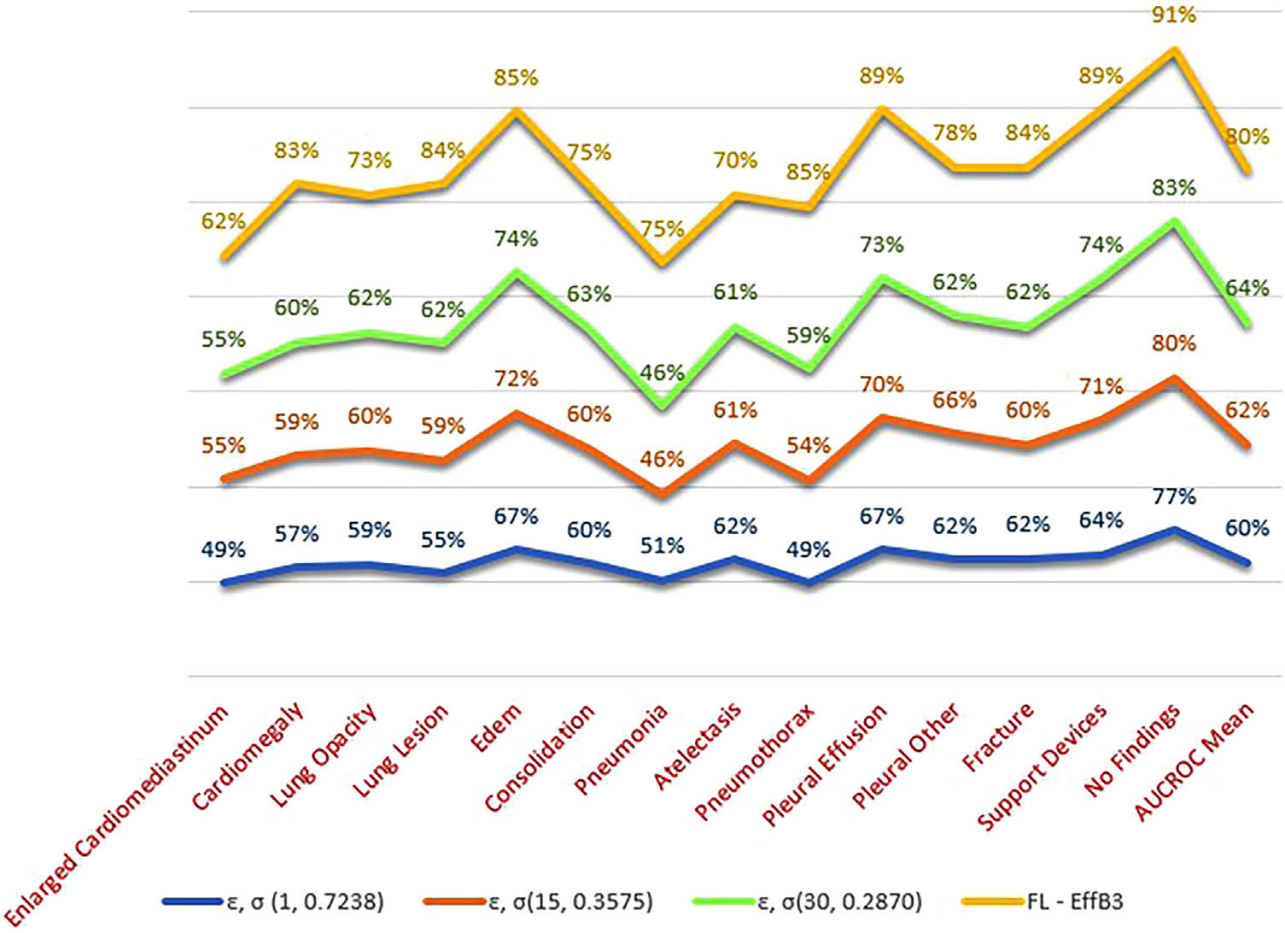
Comparison of privacy levels with FL method.

The results showed different mean AUC scores for various privacy budgets. Improving the privacy of the model can significantly impact its performance. Therefore, it is crucial to calibrate such methods to ensure that privacy enhancements do not compromise performance, and conversely, enhancing performance does not make the dataset vulnerable to attacks. The findings are summarized as follows:

- This outcome suggests that enhancing privacy can significantly reduce performance, as higher privacy settings for epsilon increase 𝜎 values, indicating more noise added to the gradients, as illustrated in Figures 6, 7, 8. The selection of clients based on the fraction value did not significantly diminish the performance of the collaboratively trained model with DP. However, using FL with DP is computationally expensive and requires substantial time and computing resources to train.
- When comparing this experiment to the proposed method in Experiment 3, which had the highest AUC of 0.8027, this experiment demonstrated lower performance with a mean AUC ranging from 0.60 to 0.6413, as shown in Figure 9.

### 4.3 Comparative Evaluation With State‐of‐the‐Art Techniques

The use of FL and FL with DP in conjunction with CNN models on the CheXpert dataset has been relatively underexplored. This insufficiency of research makes it challenging to fully understand the potential benefits and limitations of these methods in medical image analysis, particularly for chest x‐ray classification across the 14 classes in the CheXpert dataset. Comparative analysis is conducted between FL, FL with DP and more traditional centralized and decentralized FL approaches. Table 7 presents a comparative analysis of previously employed methods alongside our proposed approach. Our method demonstrates a comparative AUC mean of 0.8027 when evaluating a CNN model within an FL framework for classifying 14 thoracic classes across 10 clients. We observe improved performance as we scale up the number of clients, indicating the potential for more effective collaborative training while maintaining an acceptable performance level. Additionally, our method outperforms a few other state‐of‐the‐art models, particularly in detecting various conditions such as no finding with an AUC of 0.9066, fracture (0.8391) and lung lesion (0.8377) and is at par with other models in classes such as cardiomegaly (0.8346), pneumothorax (0.8539), support devices (0.8921) and pleural effusion (0.8939) with the 10‐client configuration. When comparing the proposed method to the baseline model, which assesses only five observations, including oedema, cardiomegaly, pleural effusion, atelectasis and consolidation, our proposed model and method demonstrate comparable performance concerning pleural effusion and oedema but show slightly lower performance in enlarged cardiomegaly, consolidation and atelectasis. A comparative analysis of ‘FL with DP’ and other state‐of‐the‐art methods reveals significant trade‐offs between privacy and performance, as illustrated in Table 7. Our earlier approach (FL with Efficient Net B3) showed comparative performance, but when compared with a more privacy‐preserving method (FL with DP), the results were significantly reduced. For a high privacy budget (𝜖 = 1, 𝜎 = 0.7238), FL with DP significantly reduces model performance, indicating that lower epsilon values can harshly impact performance, especially in medical imaging and healthcare. Thus, setting the privacy budget too low is not recommended for healthcare diagnostics.

**TABLE 7:** Comparative analysis: existing methods vs. proposed method.

Disease/Techs	Pillai [40]	Priya and Peter [41]	Chakravarty [42]	Allaouzi and Ahmed [43]	Proposed method	Proposed method (FL + DP)	𝜖, 𝜎	Irvin et al. [36]
Dataset	CheXpert	NIH x‐ray	CheXpert	CheXpert	CheXpert	CheXpert	30, 0.2870	Baseline
Model	DenseNet‐121	DenseNet‐121 + FL	CNN–GNN + FL	DenseNet‐121	EfficientNet‐B3 + FL	EfficientNet‐B3 + FL + DP		DenseNet‐121
Enlarged cardio	48.53	—	0.7563	0.71	0.6208	0.5534	30, 0.2870	0.90
Cardiomegaly	83.45	0.809	0.7204	0.88	0.8346	0.5957		
Lung opacity	91.12	—	0.8514	0.76	0.7259	0.6212		
Lung lesion	22.74	—	0.7001	0.80	0.8377	0.6189		
Oedema	88.35	0.985	0.8805	0.87	0.8466	0.7418		0.92
Consolidation	89.73	0.724	0.8077	0.77	0.7529	0.6313		0.90
Pneumonia	68.25	0.811	0.8193	0.79	0.7532	0.4648		
Atelectasis	82.43	0.882	0.7495	0.72	0.6978	0.6143		0.85
Pneumothorax	68.03	0.886	0.7915	0.86	0.8539	0.5900		
Pleural effusion	92.55	0.904	0.9047	0.90	0.8939	0.7344		0.97
Pleural other	96.99	0.583	0.8360	0.80	0.7822	0.6220		
Fracture	—	—	0.7495	0.78	0.8391	0.6211		
Support devices	88.22	—	0.9155	0.86	0.8921	0.7425		
No findings	85.01	—	0.8610	0.88	0.9066	0.8269		
**Mean AUC**	78.38	—	0.8102	0.812	0.8027	0.6413	—	—

Abbreviations: CNN, convolutional neural network; DP, differential privacy; FL, federated learning.

### 4.4 Computational Complexity Analysis

The proposed FL framework introduces additional computational and communication overhead compared to centralized learning. Each communication round requires local training at client nodes followed by aggregation at the server. The complexity increases with the number of clients and communication rounds.

Furthermore, integrating DP increases computational cost due to gradient clipping and noise injection during training. Compared to centralized models, FL with DP requires more training time but offers improved privacy guarantees.

Despite these overheads, lightweight architectures such as MobileNetV3 and EfficientNet‐B0 demonstrate better efficiency, making them suitable for deployment in resource‐constrained environments.

## 5 Discussion

The experimental findings highlight the effectiveness of the proposed FL framework for multi‐label thoracic disease classification under decentralized constraints. Unlike centralized approaches, where all data are available at a single location, FL operates under restricted data‐sharing conditions, which inherently impacts model performance. Despite these limitations, the proposed framework achieves a competitive mean AUC of 0.8027, demonstrating its capability to learn meaningful representations across distributed datasets.

A consistent trend observed across experiments is the trade‐off between model complexity and computational efficiency. Lightweight architectures such as MobileNetV3 and EfficientNet‐B0 exhibit strong performance with reduced training time, making them suitable for deployment in resource‐constrained environments. In contrast, deeper models such as DenseNet169 achieve comparable or slightly improved accuracy but at significantly higher computational cost. This highlights the importance of selecting architectures based on deployment requirements rather than accuracy alone.

The scalability analysis further indicates that increasing the number of participating clients from 5 to 10 does not significantly degrade model performance. This suggests that the FL approach can effectively generalize across multiple decentralized data sources, which is critical for real‐world healthcare applications where data are distributed across institutions.

The integration of DP introduces an expected degradation in performance due to noise injection during training. Lower privacy budgets (e.g., *ε* = 1) result in stronger privacy guarantees but significantly reduce model accuracy, whereas higher values (*ε* = 30) provide a better balance between privacy and utility. This behaviour aligns with theoretical expectations and emphasizes the importance of carefully selecting privacy parameters in sensitive domains such as healthcare.

Although transformer‐based models such as Vision Transformers (ViT) have demonstrated superior performance in centralized settings, their application in federated and privacy‐preserving environments remains challenging due to high computational requirements and sensitivity to data distribution. In contrast, this study focuses on CNN‐based architectures that offer a better balance between performance, efficiency and scalability in decentralized healthcare systems. Future work will explore hybrid CNN‐transformer models within federated settings, along with techniques for improving model interpretability and robustness.

Overall, the results emphasize that achieving the highest possible accuracy is not the sole objective in healthcare AI. Instead, practical considerations such as data privacy, scalability and deployment feasibility play a crucial role in determining the suitability of a model for real‐world applications.

## 6 Limitations

Due to the high computational cost associated with FL and DP, experiments were conducted once per configuration under controlled settings. Although this limits statistical variability analysis, consistent trends observed across multiple datasets and architectures support the reliability of the findings. Future work will include repeated trials and statistical significance testing.

Despite promising results, this study has several limitations. First, the proposed approach does not incorporate advanced architectures such as ViT, which may provide improved performance. Second, FL with DP introduces significant computational overhead and performance degradation. Third, experiments were not repeated multiple times due to computational constraints. Finally, real‐world clinical validation was not conducted, which is essential for practical deployment.

## 7 Conclusion

In this research, we embarked on the task of classifying x‐ray images across 14 classes of chest and thorax using the NIH x‐ray 14 and CheXpert datasets, using a range of CNN models. We extensively investigated different models and their outcomes to further assess CNN models for x‐ray classification within the privacy‐preserving frameworks. We aimed to determine efficient approaches to tackle challenges in medical diagnosis and contribute to advancements in this field. The selection of ResNet50, EfficientNetB0, DenseNet169 and MobileNetV3Large as our CNN models was based on established performance in image classification tasks, acquired in the literature review. To strike a balance between computer efficiency and AUC score, the tested models were compared, and an additional experiment was conducted utilizing EfficientNetB3. Throughout our analysis, we noted varying performance across different CNN architectures and datasets. Each model, including EfficientNetB0, EfficientNetB3, ResNet50, MobileNetV3Large and DenseNet169, demonstrated unique strengths across different classes and datasets, with some models excelling in specific classes in terms of AUROC score. Adjusting the hyperparameters can potentially impact the outcomes of different methods, and fine‐tuning has been employed to achieve the best possible results for these models through experimentation and refinement. Therefore, during the selection of the proposed model, consideration has been given to balancing these two aspects. Utilizing two distinct activation functions, sigmoid and Hswish, offers additional insight into their performance, with Hswish demonstrating slightly superior results. FL provides a level of security by distributing model training across decentralized devices, yet it lacks robust guarantees against privacy risks like model inversion attacks. DP enhances privacy by introducing noise to gradients or aggregated model updates, thereby safeguarding individual data contributions effectively. Achieving a balance between privacy and performance in FL with DP is critical, particularly in handling sensitive medical imaging data. Lower epsilon values, which enhance privacy, can lead to substantial performance reductions, whereas higher values may risk data security. Opting for a moderate privacy budget is advisable, necessitating careful model calibration and hyperparameter tuning. FL with DP offers a crucial trade‐off compared to traditional centralized learning, effectively safeguarding privacy while maintaining reasonable performance levels. Small institutions require both computational efficiency and moderate performance from these models, whereas larger institutions focus on better privacy mechanisms. Federated average learning proved promising and performed exceptionally well, particularly in scenarios where data privacy is indeed necessary. By employing these methods, we successfully trained robust models without centralizing data. DP proves to be a superior approach for enhancing privacy, effectively minimizing privacy risks. Our proposed experimental approach for disease classification using medical imaging data and comparative analysis highlights that our method is on par with existing methods across various diseases. EfficientB3 performed exceptionally well on the classification of 14 thoracic diseases, specifically in lung lesions with an AUC score of 0.8377 and no finding of 0.9066 in the FL environment. These findings suggest that our approach holds potential as an effective approach for disease classification in medical imaging, acceptable for further exploration and validation in clinical settings. In conclusion, our study highlights the potential of FL in medical image classification, offering a promising avenue for improving diagnostic accuracy while safeguarding patient privacy with DP.

## Author Contributions

Muhammad Zulqarnain led the conceptualization, data curation, methodology, model implementation and initial drafting. Syed Jawad Hussain and Muhammad Zeeshan Aslam contributed to data analysis and results validation, including review and editing. Ahsan Fiaz supported the literature review and experimental evaluation, with additional input on implementation and review. Muhammad Islam contributed to review and editing, model evaluation, results validation and supported the literature review and experimental evaluation.

## Funding

The authors have nothing to report.

## Conflicts of Interest

The authors declare no conflicts of interest.

## Data Availability

All data used in this study are from publicly available datasets (NIH Chest x‐ray14 and Stanford CheXpert). The corresponding source code has been made openly available at https://github.com/Zulqarnain8‐8/FEDERATED_LEARNING_FOR_THORACIC_DISEASE_CLASSIFICATION.

## References

[1] S. J. Russell and P. Norvig , Artificial Intelligence: A Modern Approach, 3rd ed. (Pearson, 2016), https://www.amazon.com/Artificial‐Intelligence‐Modern‐Approach‐3rd/dp/0136042597.

[2] M. Moshawrab , M. Adda , A. Bouzouane , H. Ibrahim , and A. Raad , “Reviewing Federated Machine Learning and Its Use in Diseases Prediction,” Sensors 23, no. 4 (2023): 2112, 10.3390/s23042112. 36850717 PMC9958993

[3] F. Chollet , Deep Learning With Python, 1st ed. (Manning, 2017), https://www.manning.com/books/deep‐learning‐with‐python‐second‐edition.

[4] A. Fiaz , B. Raza , M. Faheem , and A. Raza , “A Deep Fusion‐Based Vision Transformer for Breast Cancer Classification,” Healthcare Technology Letters 11, no. 6 (2024): 471–484, 10.1049/htl2.12093. 39720758 PMC11665795

[5] A. B. E. Ghazali , A. Fiaz , and M. Islam , “Lightweight Multi‐Stage Holistic Attention‐Based Network for Image Super‐Resolution,” IET Image Processing 19 (2025): e70013, 10.1049/ipr2.70013.

[6] M. Islam , T. Huang , E. Ahn , and U. Naseem , “Multimodal Generative AI With Autoregressive LLMS for Human Motion Understanding and Generation: A Way Forward,” preprint, arXiv:2506.03191, May 31, 2025.

[7] K. Nakayama and G. Jeno , Federated Learning With Python: Design and Implement a Federated Learning System and Develop Applications Using Existing Frameworks (Packt Publishing Ltd, 2022), https://ieeexplore.ieee.org/document/10162401.

[8] D. H. Mahlool and M. H. Abed , “A Comprehensive Survey on Federated Learning: Concept and Applications,” preprint, arXiv:2201.09384, January 23, 2022, 10.48550/arxiv.2201.09384.

[9] S. Sultana , A. Pramanik , and M. S. Rahman , “Lung Disease Classification Using Deep Learning Models From Chest X‐Ray Images,” in 2023 3rd International Conference on Intelligent Communication and Computational Techniques (ICCT) (IEEE, 2023), 10.1109/ICCT56969.2023.10075968.

[10] M. Hong , B. Rim , H. C. Lee , H. U. Jang , J. Oh , and S. Choi , “Multi‐Class Classification of Lung Diseases Using CNN Models,” Applied Sciences 11, no. 19 (2021): 9289, https://www.mdpi.com/2076‐3417/11/19/9289.

[11] H. Guan and M. Liu , “Federated Learning for Medical Image Analysis: A Survey,” preprint, arXiv:2306.05980, June 9, 2023, 10.48550/arxiv.2306.05980.

[12] P. Riedel , R. V. Schwerin , D. Schaudt , A. Hafner , and C. Späte , “Resnetfed: Federated Deep Learning Architecture for Privacy‐Preserving Pneumonia Detection From COVID‐19 Chest Radiographs,” Journal of Healthcare Informatics Research 7, no. 2 (2023): 203–224, 10.1007/s41666-023-00132-7. 37359194 PMC10265567

[13] R. I. Dumaev and S. A. Molodyakov , “Classification and Prediction of Lung Diseases According to Chest Radiography,” in 2023 IV International Conference on Neural Networks and Neurotechnologies (NeuroNT) (IEEE, 2023), 10.1109/NeuroNT58640.2023.10175838.

[14] H. B. McMahan , E. Moore , D. Ramage , S. Hampson , and B. A. Arcas , “Communication‐Efficient Learning of Deep Networks From Decentralized Data,” preprint, arXiv:1602.05629, January 26, 2016, 10.48550/arxiv.1602.05629.

[15] H. Malik , T. Anees , M. Din , and A. Naeem , “Cdc_net: Multi‐Classification Convolutional Neural Network Model for Detection of COVID‐19, Pneumothorax, Pneumonia, Lung Cancer, and Tuberculosis Using Chest X‐Rays,” Multimedia Tools and Applications 82, no. 9 (2023): 13855–13880, https://link.springer.com/article/10.1007/s11042‐022‐13843‐7. 36157356 10.1007/s11042-022-13843-7 PMC9485026

[16] A. A. Nasser and M. A. Akhloufi , “A Review of Recent Advances in Deep Learning Models for Chest Disease Detection Using Radiography,” Diagnostics 13, no. 1 (2023): 159, https://www.mdpi.com/2075‐4418/13/1/159. 36611451 10.3390/diagnostics13010159 PMC9818166

[17] A. Narin , C. Kaya , and Z. Pamuk , “Automatic Detection of Coronavirus Disease (COVID‐19) Using X‐Ray Images and Deep Convolutional Neural Networks,” Pattern Analysis and Applications 24, no. 3 (2021): 1207–1220, 10.1007/s10044-021-00984-y. 33994847 PMC8106971

[18] P. Rajpurkar , J. Irvin , K. Zhu , et al., “CheXNet: Radiologist‐Level Pneumonia Detection on Chest X‐Rays With Deep Learning,” preprint, arXiv:1711.05225, December 25, 2017, http://arxiv.org/abs/1711.05225.

[19] B. Liu , B. Yan , Y. Zhou , Y. Yang , and Y. Zhang , “Experiments of Federated Learning for COVID‐19 Chest X‐Ray Images,” preprint, arXiv:2007.05592, July 5, 2020, 10.48550/arxiv.2007.05592.

[20] Z. Wang , K. Zhang , and B. Wang , “Detection of COVID‐19 Cases Based on Deep Learning With X‐Ray Images,” Electronics 11, no. 21 (2022): 3511, 10.3390/electronics11213511.

[21] E. Khan , M. Z. U. Rehman , F. Ahmed , F. A. Alfouzan , N. M. Alzahrani , and J. Ahmad , “Chest X‐Ray Classification for the Detection of COVID‐19 Using Deep Learning Techniques,” Sensors 22, no. 3 (2022): 1211, 10.3390/s22031211. 35161958 PMC8838072

[22] E. Ayan and H. M. Ünver , “Diagnosis of Pneumonia From Chest X‐Ray Images Using Deep Learning,” in 2019 Scientific Meeting on Electrical‐Electronics & Biomedical Engineering and Computer Science (EBBT), Istanbul, Turkey (2019), 10.1109/EBBT.2019.8741582.

[23] A. Vij and K. S. Kaswan , “Prediction of Lung Cancer Using Convolution Neural Networks,” in 2023 International Conference on Artificial Intelligence and Smart Communication (AISC) (IEEE, 2023), 10.1109/AISC56616.2023.10085058.

[24] S. Liu , “Enhancing Breast Cancer Classification Using Transfer Resnet With Lightweight Attention Mechanism,” preprint, arXiv:2308.13150v2, December 23, 2023, https://arxiv.org/abs/2308.13150v2.

[25] A. M. Q. Farhan and S. Yang , “Automatic Lung Disease Classification From the Chest X‐Ray Images Using Hybrid Deep Learning Algorithm,” Multimedia Tools and Applications 82 (2023): 38561–38587, 10.1007/s11042-023-15047-z. PMC10030349 37362647

[26] J. Zhao , M. Li , W. Shi , Y. Miao , Z. Jiang , and B. Ji , “A Deep Learning Method for Classification of Chest X‐Ray Images,” Journal of Physics: Conference Series 1848 (2021): 012030, 10.1088/1742-6596/1848/1/012030.

[27] S. K. Vanga , V. Gourishetty , A. Shetkar , and P. Yadlapalli , “Multi Lung Disease Detection,” in 2023 4th International Conference for Emerging Technology (INCET), Belgaum, India (2023), 10.1109/INCET57972.2023.10170401; https://arxiv.org/pdf/2110.04160.

[28] K. V. Priya and J. D. Peter , “A Federated Approach for Detecting the Chest Diseases Using DenseNet for Multi‐Label Classification,” Complex and Intelligent Systems 8, no. 4 (2022): 3121–3129, 10.1007/s40747-021-00474-y.

[29] S. J. Pan and Q. Yang , “A Survey on Transfer Learning,” IEEE Transactions on Knowledge and Data Engineering 22, no. 10 (2010): 1345–1359, 10.1109/TKDE.2009.191.

[30] A. A. Khan , B. Hussain , M. Islam , M. M. Al Dabel , and A. K. Bashir , “Optimizing Content Cache With Vehicular Edge Computing: A Deep Federated Learning Based Novel Predictive Study,” IEEE Transactions on Consumer Electronics 71, no. 2 (2025): 6069–6079, 10.1109/TCE.2025.3571029.

[31] H. Tran and Y. Huang , “Federated SGD COVID‐19 Detection Under Local Differential Privacy Using Chest X‐Ray Images and Symptom Information,” Sensors 22, no. 10 (2022): 3728, 10.3390/s22103728. 35632136 PMC9147951

[32] A. Rehman , A. Khan , G. Fatima , S. Naz , and I. Razzak , “Review on Chest Pathologies Detection Systems Using Deep Learning Techniques,” Artificial Intelligence Review 56 (2023): 12607–12653, 10.1007/s10462-023-10457-9. PMC10027283 37362896

[33] S. Jaeger , S. Candemir , S. Antani , Y.‐X. J. Wáng , P. Lu , and G. Thoma , “Two Public Chest X‐Ray Datasets for Computer‐Aided Screening of Pulmonary Diseases,” Quantitative Imaging in Medicine and Surgery 4, no. 6 (2014): 475–477, 10.3978/j.issn.2223-4292.2014.11.20. 25525580 PMC4256233

[34] D. Demner‐Fushman , W. J. Rogers , A. R. Aronson , et al., “Preparing a Collection of Radiology Examinations for Distribution and Retrieval,” Journal of the American Medical Informatics Association 23, no. 2 (2016): 304–310, 10.1093/jamia/ocv080. 26133894 PMC5009925

[35] X. Wang , Y. Peng , L. Lu , Z. Lu , M. Bagheri , and R. M. Summers , “Chestx‐Ray8: Hospital‐Scale Chest X‐Ray Database and Benchmarks on Weakly‐Supervised Classification and Localization of Common Thorax Diseases,” in Proceedings of the IEEE Conference on Computer Vision and Pattern Recognition (CVPR) (IEEE, 2017).

[36] J. Irvin , P. Rajpurkar , M. Ko , et al., “CheXpert: A Large Chest Radiograph Dataset With Uncertainty Labels and Expert Comparison,” Proceedings of the AAAI Conference on Artificial Intelligence 33 (2019): 590–597, 10.1609/aaai.v33i01.3301590.

[37] A. E. W. Johnson , T. J. Pollard , N. R. Greenbaum , et al., “Mimic‐CXR, a De‐Identified Publicly Available Database of Chest Radiographs With Free‐Text Reports,” Scientific Data 6, no. 1 (2019): 317, 10.1038/s41597-019-0322-0. 31831740 PMC6908718

[38] S. Singh , S. S. Rawat , M. Gupta , et al., “Deep Attention Network for Pneumonia Detection Using Chest X‐Ray Images,” Computers, Materials Continua 74, no. 1 (2023): 1673–1691, 10.32604/cmc.2023.032364.

[39] F. S. Nahm , “Receiver Operating Characteristic Curve: Overview and Practical Use for Clinicians,” Korean Journal of Anesthesiology 75, no. 1 (2022): 25–36, 10.4097/kja.21209. 35124947 PMC8831439

[40] A. S. Pillai , “Multi‐Label Chest X‐Ray Classification via Deep Learning,” Journal of Intelligent Learning Systems and Applications 14, no. 4 (2022): 43–56, 10.4236/jilsa.2022.144004.

[41] K. V. Priya and J. D. Peter , “A Federated Approach for Detecting the Chest Diseases Using DenseNet for Multi‐Label Classification,” Complex & Intelligent Systems 8 (2022): 3121–3129, 10.1007/s40747-021-00474-y.

[42] A. Chakravarty , A. Kar , R. Sethuraman , and D. Sheet , “Federated Learning for Site Aware Chest Radiograph Screening,” in 2021 IEEE 18th International Symposium on Biomedical Imaging (ISBI), Nice, France (IEEE, 2021), 10.1109/ISBI48211.2021.9433876.

[43] I. Allaouzi and M. B. Ahmed , “A Novel Approach for Multi‐Label Chest X‐Ray Classification of Common Thorax Diseases,” IEEE Access 7 (2019): 64279–64288, 10.1109/ACCESS.2019.2916849.

[44] L. Alzubaidi , J. Bai , A. Al‐Sabaawi , et al., “A Survey on Deep Learning Tools Dealing With Data Scarcity: Definitions, Challenges, Solutions, Tips, and Applications,” Journal of Big Data 10, no. 1 (2023): 46, 10.1186/s40537-023-00727-2.

